# Sublethal Doses of Anthrax Lethal Toxin on the Suppression of Macrophage Phagocytosis

**DOI:** 10.1371/journal.pone.0014289

**Published:** 2010-12-10

**Authors:** Jyh-Hwa Kau, Der-Shan Sun, Hsuan-Shun Huang, Te-Sheng Lien, Hsin-Hsien Huang, Hung-Chi Lin, Hsin-Hou Chang

**Affiliations:** 1 Institute of Preventive Medicine, National Defense Medical Center, Taipei, Taiwan, Republic of China; 2 Department of Molecular Biology and Human Genetics, Tzu-Chi University, Hualien, Taiwan, Republic of China; Columbia University, United States of America

## Abstract

**Background:**

Lethal toxin (LT), the major virulence factor produced by *Bacillus anthracis,* has been shown to suppress the immune system, which is beneficial to the establishment of *B. anthracis* infections. It has been suggested that the suppression of MEK/MAPK signaling pathways of leukocytes contributes to LT-mediated immunosuppressive effects. However, the involvement of MAPK independent pathways has not been clearly elucidated; nor has the crucial role played by LT in the early stages of infection. Determining whether LT exerts any pathological effects before being enriched to an MEK inhibitory level is an important next step in the furtherance of this field.

**Methodology/Principal Findings:**

Using a cell culture model, we determined that low doses of LT inhibited phagocytosis of macrophages, without influencing MAPK pathways. Consistent low doses of LT significantly suppressed bacterial clearance and enhanced the mortality of mice with bacteremia, without suppressing the MEK1 of splenic and peripheral blood mononuclear cells.

**Conclusion/Significance:**

These results suggest that LT suppresses the phagocytes in a dose range lower than that required to suppress MEK1 in the early stages of infection.

## Introduction

Anthrax refers to diseases caused by infection with the Gram-positive bacterium *Bacillus anthracis*. The bacteria replicates to very high numbers in the blood, leading to the death of the host. Because the bacteria spread extensively without an evident immune response, it has been suggested that the pathogen impairs the immune system of the host. Recent studies have shown that lethal toxin (LT), the major virulence factor of *Bacillus anthracis* [1]–[3], strongly contributes to this intervention through pleiotropic action on various cells of the host immune system [4]–[5]. Lethal toxin isogenic mutant bacteria lacks single toxin components and is therefore greatly attenuated [6]–[7]. Lethal toxin is a bipartite protein complex comprising protective antigen (PA) and the lethal factor (LF), which functionally inactivates the mitogen activated protein kinase kinases (MAPKKs/MEKs) through metalloprotease activity of LF [8]–[10]. Mitogen activated protein kinases (MAPKs) are downstream effectors of MEKs, which were divided into the extracellular-regulated kinase (ERK, p42/44), p38, and c-Jun amino terminal protein kinase (JNK) subgroups. These have been implicated in multiple cellular events such as proliferation, survival, differentiation and inflammation [11]. Lethal toxin inhibits phagocytic function through the suppression of MEK-mediated generation of bactericidal superoxide and MEK-mediated survival of macrophage cells [12]–[13]. Whether or not LT suppresses macrophage function through a MEK/MAPK independent pathway, however, remains unclear.

Some evidences have implied that LT might play an inhibitory role in phagocytic function of phagocytes at low doses without perturbing the MEK/MAPK pathways. Lethal toxin was shown to inhibit actin dynamics without influencing the MEK1 pathway; the inhibition of actin dynamics blocks the migration of neutrophil and *Listeria*-driven actin motility [14]. This implies that LT-mediated inhibition might also affect on phagocytosis, an actin dependent process. Phagocytic function of phagocytes is important for the clearance of bacteria. This was indicated by another major virulence factor of *B. anthracis*, the poly-γ-_D_-glutamic acid capsule, inhibiting phagocytosis through nonimmunogenic surfaces, which is critical for full virulence [15]. It has also been shown that *in vivo* treatments for LT reduced fluorescent polystyrene beads engaged with mouse spleen macrophages [16], and may likely support the suppressive role of LT on phagocytosis. However, treatment under the same conditions also suppressed the MEK-C/EBP axis signaling pathways and induced apoptosis of spleen macrophages [16]. This leads to the conclusion that an MEK-dependent process is still involved, while the involvement of MEK-independent pathway remains unclear. As the role of LT in the early phases of infection is an important issue deserving of further investigations [17], it would be interesting to address the potential pathological role of low dose LT, by which it would not influence the MEK pathway. As a result, in this study we sought to investigate the inhibitory role of low dose LT on phagocytosis. Related issues regarding whether or not this suppression on phagocytes is beneficial for the survival of infected bacteria are also discussed.

## Results

### Various doses of LT affected cell survival, MEK/MAPK pathways and phagocytosis of macrophage cells

**Figure 1:**
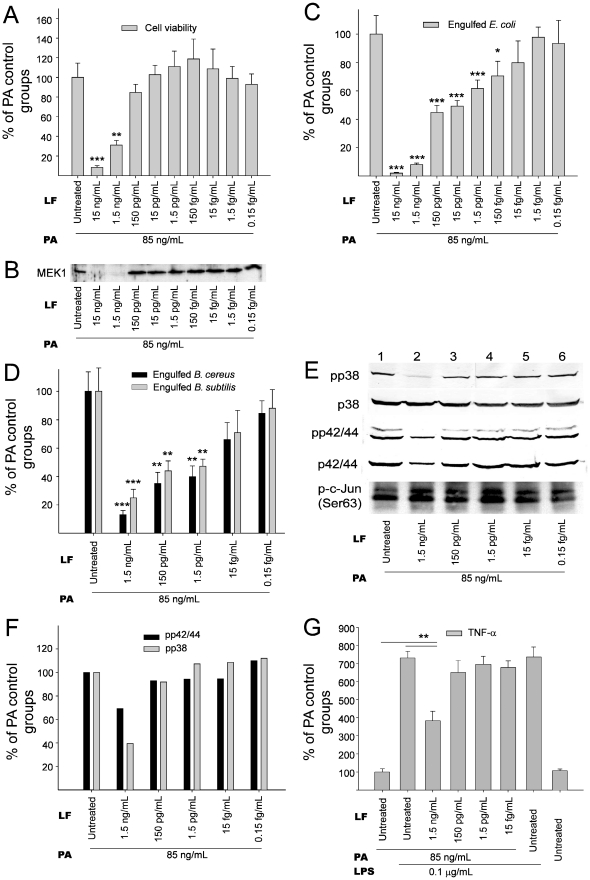
LT-mediated suppression of macrophage cells. Inhibitory effects of various concentrations of LT treatments (3 hours) on cell viability (A), intact MEK1 levels (B), phagocytosis of *E. coli* (C), *B. cereus* and *B. subtilis* (D), MAPK pathways (E, F) and TNF-α production (G) of mouse macrophage J774A.1 cells are shown. The numbers of surviving cells (A) and surviving bacteria after phagocytosis (C, D) in the PA groups (LF untreated) were adjusted to 100%. Levels of pp38, p38, pp42/44, p42/44 MAPKs in J774A.1 cells treated with LPS and LT were analyzed by Western blotting (E). Phosphorylated c-Jun (p-c-Jun) levels revealed JNK activity in J774A.1 cells (E). Quantitative results of pp42/44 and pp38 levels were obtained by measuring gel intensity normalized with internal controls p42/44 and p38, respectively (F); the levels in the PA groups (LF untreated) were adjusted to 100%. After various PA, LT and LPS treatments, the level of TNF-α secreted by J774A.1 cells was determined by ELISA (G); PA groups (without LF and LPS treatment) were adjusted to 100%. Error bars indicate standard deviations. * *P*<0.05, ** *P*<0.01, *** *P*<0.001 compared to the PA controls (PA: 85 ng/mL). n = 6 (3 experiments with 2 replicates) (A, C, D, G).

The dosage effect of LT on the suppression of cell viability and intact MEK1 levels was analyzed using J774A.1 cells (Fig. 1A, B), a mouse macrophage cell line commonly applied for the study of LT function [13], [18]. We found that the suppression on MEK1 is associated with the induction of cell death when treated with various doses of LT (Fig. 1A, 1B; significant suppression in 1.5 and 15 ng/mL LF groups vs. no significant suppression in the other groups). The phagocytosis of living bacteria *Escherichia coli*, *Bacillus cereus* and *Bacillus subtilis*, which were used as surrogates as *B. anthracis*, were then analyzed under treatments with LT in the same range of dosage (Fig. 1C, 1D). Our data revealed that LT significantly suppressed phagocytic activity, regardless of the bacterium used (Fig. 1C, D), suggesting that such suppression occurred without strain specificity. Intriguingly, the effective dose of LT on phagocytosis includes low doses that do not significantly suppress either cell survival or MEK1 (Fig. 1C and 1D vs. 1A and 1B, 1.5–150 pg/mL LF groups). In addition, MEK1, LT also blocks other MEKs [19]. To further investigate how LT treatments influence MAPKs, the downstream of MEKs, we performed Western blotting analysis (Fig. 1E). Respective gel densities of the phospho-p38 (pp38) MAPK and the phospho-p42/44 (pp42/44) MAPK under various treatments are shown (Fig. 1F). Our data indicated that LT primarily blocked the activation of p38 MAPK but not the p42/44 MAPK and JNK under treatment with lipopolysaccharide (LPS) (Fig. 1E, 1F; 1.5 ng/mL LF). Intriguingly, here we found that the inhibitory dose of LT on p38 MAPK (Fig. 1E, 1.5 ng/mL LF) was concurrent with the doses that affect both cell survival (Fig. 1A) and MEK1 (Fig. 1B). As LPS-induced cytokine production of macrophage was largely blocked when MAPKs were inactivated [20]–[21], we found that the inhibitory dose of LT for TNF-α production (Fig. 1G, 1.5 ng/mL LF groups) was also consistent with that of the cell viability, MEK1 and p38MAPK data (Fig. 1A, 1B, 1E, 1.5 ng/mL LF groups). These results suggest that LT influenced MEK1 and MEK3/MEK6, the upstream of p38 MAPK, in a similar dose range in murine macrophages. In addition, such suppression of phagocytosis could be induced by low dose LT without influencing the MAPK pathways or cell survival.

### Flow cytometry analyses of cell death and phagocytosis of fluorescent beads

**Figure 2:**
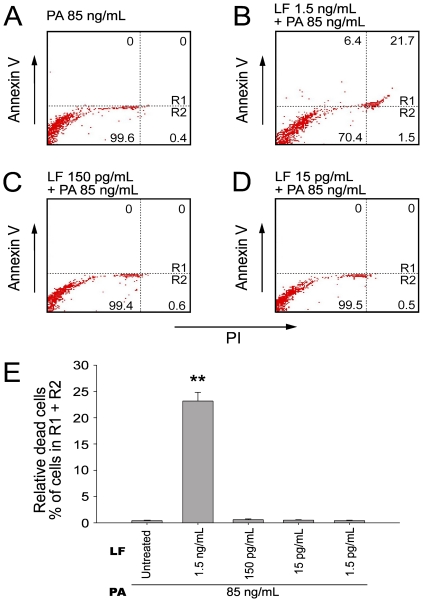
Flow cytometry analysis for LT-induced cell death of macrophage cells. Flow cytometry analysis was employed to determine the cell death of macrophage cells (A–D). After 3-hour treatments of various concentrations of LT, mouse macrophage J774A.1 cells were stained with annexin V-APC and PI. The upper right quadrants (Rl) and the lower right quadrants (R2) were positive for PI uptake representing the non-viable cells (A–D). One representative diagram out of three experiments is showed, in which the percentage of cells in each quadrant was indicated (A–D). The quantitative results of relative dead cells (% of cell subpopulations in R1 + R2) are presented as mean ± standard deviation (E). ** *P*<0.01, compared to the PA control groups (LF untreated).

**Figure 3:**
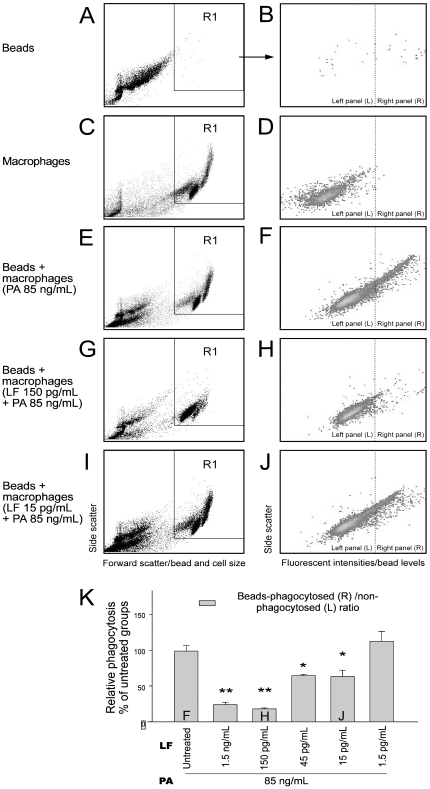
Flow cytometry analysis for phagocytosis. Flow cytometry analysis was employed to determine the phagocytosed level of fluorescent beads by macrophages (A–J). The time course of LT and PA treatments was 3 hours. In panels A, C, E, G, I, the X-axis represents particle/cell size (forward scatter) and the Y-axis represents the granularity of cells (side scatter). A and B: fluorescent beads only, C and D: macrophage cells only, E and F: beads + PA (85 ng/mL) treated cells, G and H: beads + LT (LF 150 pg/mL + PA 85 ng/mL) treated cells, I and J: beads + LT (LF 15 pg/mL + PA 85 ng/mL) treated cells. One representative diagram out of three experiments is shown (A–J). The R1 region in panels A, C, E, G and I reveals the population of the macrophage, which was specifically gated to obtain the graphs B, D, F, H, J, respectively. In panels B, D, F, H, J, the X-axis represents fluorescent intensity, which indicates the levels of macrophage-engaged beads, and the Y-axis represents the granularity of the cells (side scatter). The percentage of the cells with relatively high fluorescence intensity on the right panels of B, D, F, H, J, indicated the phagocytosed levels of beads by macrophages. In contrast, the percentage of cells on the left panels with relatively low fluorescence intensity indicates the number of cells without phagocytosed beads (B, D, F, H, J). The relative phagocytosis of macrophages was quantified by calculating the beads-phagocytosed (right panel: R)/non-phagocytosed (left panel: L) ratio of macrophages (K), the level of LF untreated (PA only) groups were normalized to 100%. The F, H, J labels in the columns of Fig. 3K indicate the respective conditions as showed in Fig. 3F, 3H and 3J, respectively (K). Error bars indicate standard deviations of three experiments. * *P*<0.05, ** *P*<0.01 compared to the PA (LF untreated) control groups. n = 6 (3 experiments with 2 replicates).

We employed flow cytometry to determine whether LT-treated cells were actually killed by the toxin, and, therefore, unable to phagocytose the bacteria. Through annexin V and propidium iodide (PI) staining, we found that the LT treatments could only induce cell death in the 1.5 ng/mL LF groups but not the other groups treated with lower doses of LT (Fig. 2; 2B vs. 2C, 2D; 2E, 1.5 ng/mL LF groups vs. 1.5–150 pg/mL groups). This is consistent with our WST-1 data (Fig. 1A, 1.5 ng/mL LF groups), and further indicates that the suppression of phagocytosis by lower doses of LT was not likely caused by cell death. In the above phagocytosis experiments (Fig. 1C, 1D), we measured phagocytosis using a gentamicin protection assay [22]–[26]. After plating the macrophage lysate on agar plates, the LT treated groups with low *E. coli* counts were considered to exhibit low phagocytosis activity. Low *E. coli* counts, however, might be explained by an enhanced killing of the engulfed *E. coli* in LT-activated macrophages rather than a lower uptake of these bacteria, or alternatively, increased permeability of LT-treated cells to the antibiotic gentamicin. To confirm the suppressive effect of LT, fluorescent beads were used as phagocytosis targets and analyzed using flow cytometry (Fig. 3). Macrophage J774A.1 cells were allowed to engulf immunoglobulin-opsonized fluorescent beads. We were able to distinguish the beads from the macrophages using particle/cell-size differences (Fig. 3A vs. 3C, 3E, 3G; 3I; R1 regions: J774A.1 cells). By differentiating the fluorescent signal of cells, we were able to measure the beads-phagocytosed phagocytes of PA and LT treated groups (Fig. 3F, 3H, 3J, right panels). Relative phagocytosis was determined by calculating the beads- phagocytosed/non-phagocytosed ratio of the cell population (Fig. 3K). We found that treatments using doses higher than 15 pg/mL of LF (with 85 ng/mL PA) significantly reduced the engulfment on beads (Fig. 3K, * *P*<0.05, ** *P*<0.01, 15 pg/mL LF groups vs. LF untreated groups), which is partly consistent with our phagocytosis data using living bacteria (Fig. 1C, 1D, 15 and 150 pg/mL LF groups). When the kinase data was also taken into consideration (Fig. 1B and 1E, no inhibition in 15 and 150 pg/mL LF groups), these results suggested that low dose LT-mediated suppression on phagocytosis involving a MAPK independent pathway.

### Low plasma levels of LT detected in mice in early stages of infection

**Figure 4:**
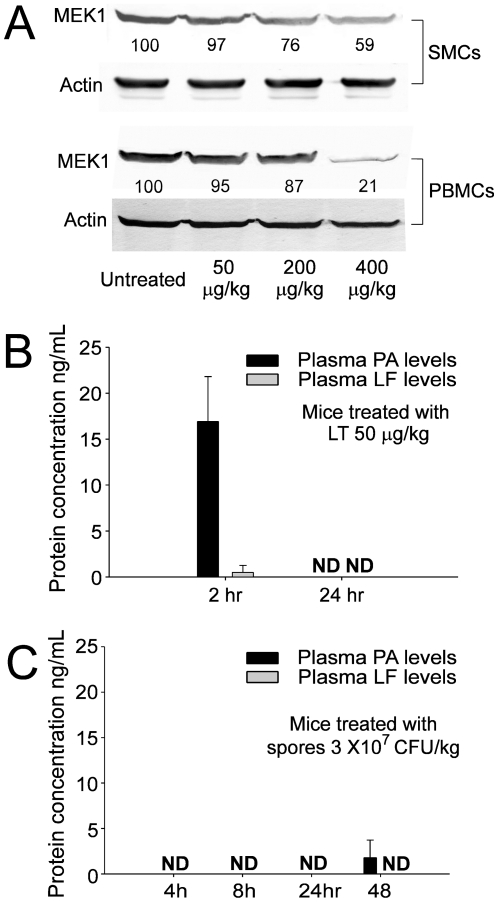
Leukocyte MEK1 levels and plasma LT levels in LT treated mice. After mice were treated with various doses (50, 200 and 400 µg/kg) of LT intravenously for 3 hours, intact MEK1 levels of mouse splenic mononuclear cells (SMCs) and peripheral blood mononuclear cells (PBMCs) were analyzed using Western blotting (A). Cellular actin levels were used as internal controls of respective groups (A). The relative gel densities after being normalized with respective actin levels are shown; the level of untreated groups was adjusted to 100% (A). The plasma PA and LF levels were determined using ELISA from specimens of mice treated with LT (50 µg/kg, a sublethal dose) (B), or spores of *B. anthracis* (3×10^7^ CFU/kg, a lethal dose) (C). ND: not detectable. n = 6 (3 experiments with 2 replicates) (B, 2 hr groups; C). n = 10 (3 experiments with 3 or 4 replicates) (B, 24 hr groups).

To analyze the effects of low doses of LT on the suppression of phagocytosis *in vivo*, we examined the MEK1 level of mouse splenic mononuclear cells (SMCs) and peripheral blood mononuclear cells (PBMCs) after treatment with LT. Our data revealed that intravenous LT treatments with doses of 200 and 400 µg/kg, (but not 50 µg/kg) (LF∶PA = 15∶85) significantly suppressed the MEK1 level of SMCs and PBMCs of the mice (Fig. 4A). The corresponding mouse plasma levels of PA and LF within two and 24 hours following treatments with LT (50 µg/kg) are shown (Fig. 4B). The concentration of LF was much lower than the level required to suppress MEK1 and MAPK pathways *in vitro* (Fig. 4B LF levels: <1 ng/mL in 2 hr groups, and not detectable [ND] in 24 hr groups vs. Fig. 1B, 1E 1.5 ng/mL groups). Theoretically, the effective dosage of LT in the early infectious stages is crucial to the survival of the bacterium. As a result, mouse plasma PA and LF concentrations in the early stages of infection were also examined. Mice in the experiment were treated with a lethal dose of *B. anthracis* spores (3×10^7^ CFU/kg; lethality began at 48–72 hours after the treatment), and the plasma levels of PA and LF were measured at various points in time. Our data revealed that during the first 24 hours, PA and LF were not detectable in the plasma of mice; 48 hours following treatment, only a low level of PA was barely detectable. The low level of PA and LF detected in mice during the early stages of the infection was somewhat similar to a previous report using other experimental animals [27]. Our data implies that the plasma level of LT in the early stages of infection was much lower than the MEK1/MAPK suppressive levels under both *in vitro* and *in vivo* conditions (Fig. 1B and 1E 1.5 ng/mL LF groups vs. 4A and 4B 50 µg/kg LT groups vs. 4C 48 hrs groups).

### Low dose of LT suppressed the bacterial clearance and enhanced mortality in septic mice

**Figure 5:**
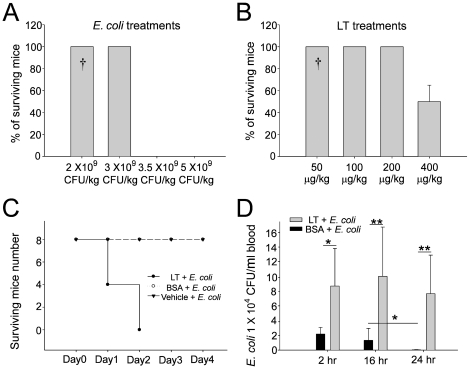
Treatment with sublethal doses of LT enhanced mortality and impaired bacterial clearance in mice with bacteremia. To test the sublethal dose of LT, mortality in C57BL/6J mice was measured after treatment with different doses of *E. coli* (A, n = 8) or LT (B, n = 8) (4 experiments with 2 replicates). The mortality of mice with *E. coli* bacteremia could be enhanced by sub-MAPK LT treatments (50 µg/kg), but not by equivalent quantities of control protein BSA or vehicle treatments (C, n = 8) (4 experiments with 2 replicates). The surviving *E. coli* in the plasma of the septic mice was enhanced by the treatment of sublethal doses of LT (50 µg/kg) (D, n = 6) (3 experiments with 2 replicates). The **^†^** marks shown in A and B indicates the dosages of *E. coli* and LT that used in C and D. * *P*<0.05, ** *P*<0.01.

According to our results, sub-MEK1 inhibitory doses of LF (15 pg/mL to 150 pg/mL) plus adequate PA (85 ng/mL) are sufficient to suppress the macrophage phagocytosis *in vitro* (Fig. 1C, 1D, 3K). Theoretically, such low doses of LT might be sufficient to enhance the mortality and bacterial survival in septic mice. As a result, a septic shock mouse model was employed. First, we measured the sublethal and lethal doses of *E. coli* and LT, respectively. We found that treatment using *E. coli* at doses higher than 3.5×10^9^ CFU/kg, or using LT doses higher than 400 µg/kg could induce mortality in mice (Fig. 5A, 5B). The LT dose 50 µg/kg is not only a sublethal dose for mice (Fig. 5B), but also a sub-MEK1 inhibitory dose of mouse splenic and peripheral blood mononuclear cells (Fig. 4A, SMCs and PBMCs groups). Notably, even though sublethal treatments using either *E. coli* (Fig. 5A, 2×10^9 ^CFU/kg) or LT (Fig. 5B, 50 µg/kg) alone showed no mortality, and a combination of both treatments resulted in 100% mortality among the mice (Fig. 5C). In contrast, combined treatments using vehicle or control protein bovine serum albumin (BSA) plus *E. coli* (2×10^9^ CFU/kg) did not lead to mortality (Fig. 5C). To investigate the inhibitory role of sub-MEK1 dose of LT (Fig. 4A, 50 µg/kg) on the bacterial clearance *in vivo*, viable bacteria (CFU) in mouse blood specimens were analyzed after combined treatment with LT and *E. coli* for 2, 16 and 24 hours (Fig. 5D). The control mice treated with BSA significantly eliminated bacteria from their circulation within 24 hours, while LT-treated mice showed no obvious clearance during the same period, as evidenced by the 4–6 fold higher levels of residual surviving bacteria in their circulation (Fig. 5D, LT vs. BSA groups; * *P*<0.05, ** *P*<0.01). These findings suggested that LT-mediated suppression with a sub-MEK1 inhibitory dose of splenic and peripheral blood mononuclear cells is sufficiently beneficial for bacteria to survive host clearance.

## Discussion

Phagocytosis is a process relying highly on actin cytoskeletal dynamics [28]. Specific mechanisms that block actin dynamics, in so doing suppress phagocytic uptake into macrophages. Such mechanisms are evolved by various pathogens to avoid robust bactericidal effector functions [29]. Given that the MEK/MAPK pathways play an important role in the regulation of cytoskeletal dynamics [30], and that LT, an MEK inhibitor, could block actin dynamics [14], [31], it is not surprising to observe the inhibitory role of LT on phagocytosis. However, whether or not there involves a MAPK-independent pathway in LT-mediated suppression on phagocytosis, is not clearly demonstrated. Intriguingly, our data suggested that such inhibitory pathways are likely to exist.

**Figure 6:**
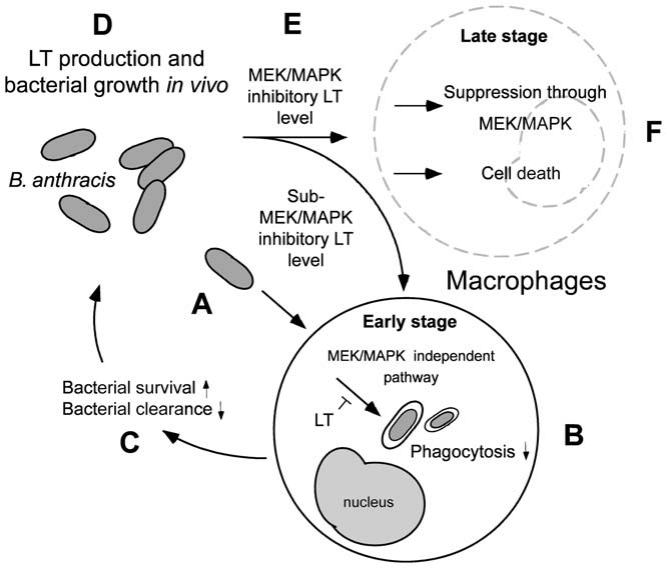
A hypothetical model. In the early infectious stage of *B. anthracis* (A), LT likely suppresses phagocytosis at a low level resulting in an increase in bacterial survival and decrease in host clearance without influencing the MEK/MAPK pathways (B–C). As the surviving bacteria accumulated to a high level (D), the LT level may gradually reach to a MAPK inhibitory dose (E) leading to further disruption of host homeostasis and immune responses (F). ↓ Activation processes. ⊥ Inhibition processes.

Using a cell culture model, here we find that LT could suppress the phagocytosis of J774A.1 macrophage cells at low doses without influencing the MAPK pathways (Fig. 1C, 1D and 3K; 15 pg/mL groups). A septic shock mouse model revealed that the anti-phagocytic properties of LT could exacerbate the bacterial clearance and enhance mortality in mice (Fig. 5C). Enhanced mortality in septic animal after LT treatment is to a certain degree consistent with a previous study [32]. These authors shed light on the anti-inflammatory role of LT on the amelioration of LPS treatment in mice, while the exacerbation of LT on *E. coli* treated mice, however, was not specifically linked to the suppression of macrophage phagocytosis [32]. Our data provides an alternative explanation in which such phenomena could be attributed to the suppression of phagocytosis by LT, because LT greatly reduced bacterial clearance (Fig. 5D). Because we used low doses of LT without influencing the MEK1 of splenic and peripheral blood mononuclear cells (Fig. 4A, 50 µg/kg LT groups), the MEK-dependent suppression on bactericidal superoxide [12] is not likely to be involved. Because phagocytic cells play an important roles in the cross road of innate immunity and adaptive immunity [33], our findings may broaden our current understanding of the immunosuppressive properties of LT. It is well known that LT paralyzes the immune system primarily mediated through the inhibition of MEK/MAPK pathways, and in part mediated through the initiation of leukocyte cell death [5], [17], [34]. Intriguingly, here we found that the phagocytic inhibitory doses of LT could be far lower than that of eliciting cell death of phagocytes (Fig. 1A, 2E vs. 1C, 1D, 3K), and that such inhibition did not significantly influence the MEK/MAPK pathways (Fig. 1B, 1E, vs. 1C, 1D, 3K). A reasonable speculation is that the immunosuppressive effect of LT might be initiated at a relatively earlier infectious stage, in which LT is produced at low doses. Accordingly, here we proposed a hypothetical model (Fig. 6). During the early infections, *B. anthracis* expressed low levels of LT that are insufficient to suppress the MEK pathway of phagocytes (Fig. 4A, 4C), but critical for the inhibition of phagocytic clearance of bacterial pathogens (Fig. 5D; 6A–C). After *B. anthracis* is amplified to a greater population in the host (Fig. 6D), the LT gradually reached to an MEK/MAPK inhibitory dose (Fig. 6E) and finally led to the suppression of MEK/MAPK and cell death of macrophages (Fig. 6F). The molecular target of LT under such low doses, however, requires further investigation.

One of the candidate pathways is through NALP1. It has been shown that the susceptibility of different mouse strains is linked to the polymorphic locus *Nalp1b* [35]. The product of this locus, NALP1, is a component of inflammasone, a protein complex consisting of NALP1 and caspases 1, leading to the processing and release of pro-inflammatory cytokines IL-1β and IL-18 [36]. Activation of caspase-1 by NALP1 mediates murine macrophage cytolysis through the mitochondrial protein Bnip3 and Bnip3L [35], [37]. Because MEK cleavage occurs in both resistant and susceptible cells [13], [38], but NALP1 activation occurs in susceptible macrophages only [35], these evidences suggest that LT-mediated NALP1 suppression might be independent of MEK pathways. However, NALP1 is linked to LT-mediated suppression of other cellular functions, while the phagocytosis has not been characterized in these studies. As a result, it may be suggested that NALP1 or a yet unidentified pathway is responsible for the suppression of phagocytosis by low dose LT. Further investigations are required to characterize the specific pathways involved.

In summary, here we demonstrated that LT suppresses macrophage phagocytosis through both MEK/MAPK dependent and independent pathways. Our results suggest that a two-step inhibition of macrophage phagocytosis by LT. The first stage involved a MEK-independent inhibition when the LT concentration was lower than the MEK/MAPK suppressive dosage (Fig. 6B). In this stage, LT suppresses macrophage phagocytosis and bacterial clearance *in vivo* to facilitate bacterial infection (Fig. 6C). Because anthrax can cause massive bacteremia [34], in the second stage, LT might progressively increase and eventually reach an MEK/MAPK suppressive dose, thereby leading to a p38 MAPK dependent inhibition (Fig. 6D-F). Our results suggest that LT is able to transmit a phagocytic suppressive signal through an MEK/MAPK independent pathway, which could be beneficial for the survival of *B. anthracis* during early infectious stages.

## Materials and Methods

### Ethics Statement

The research methods applied regarding the experimental mice were approved by the Animal Care and Use Committee of Tzu Chi University (approval ID: 95017 and 98104).

### Mice, bacteria and toxin

The C57BL/6J mice (males, 8-11 weeks of age) were purchased from National Laboratory Animal Center (NLAC, Taipei, Taiwan). Mice were housed in the Laboratory Animal Center of Tzu Chi University (Hualien, Taiwan). B. anthracis (ATCC 14186), which contains both pXO1 and pXO2 plasmids that express functional lethal toxin (LT) and edema toxin (ET), was grown and maintained as previously described [18], [39]. Native *B. anthracis* lethal toxins, PA and LF, were purified from the culture supernatants of *B. anthracis* (ATCC 14186). The bacteria *E. coli* (OP50, a laboratory strain [40]–[41]), *B. cereus* (ATCC 7004) and *B. subtilis* (ATCC 21336) were used in the phagocytosis experiments.

### Cell viability analysis

The mouse macrophage-like cell line J774A.1 (ATCC TIB67) was maintained in Dulbeco's modified Eagle's medium (DMEM) containing 10% fetal bovine serum (FBS) used in cytotoxicity analysis [18], [26]. After being changed with serum free medium, cells (1×10^5^/well) were treated with different concentrations (0.15 fg/mL to 15 ng/mL) of LF plus 85 ng/mL PA in a 96-well cell culture dish. Three hours after treatments, the level of viable cells was analyzed using the WST-1 kit (Roche Diagnostics, Taipei, Taiwan) according to the manufacturer's instructions [26], [42]–[43]. The assay principle was based on a reduction of the tetrazolium salt WST-1 to formazan by cellular dehydrogenases in viable cells. The generation of yellow formazan was measurable at 450 nm and was correlated to the number of cells. A standard curve plotted between WST-1 values with serious diluted J774A.1 cells (0 to 5×10^5^) was obtained first, and the relative number of viable cells was then calculated, accordingly.

### Phagocytosis analysis by gentamicin protection assay

Gentamicin protection assay were performed as described in previous literatures [24]–[25], [44]–[45]. Live bacteria *E. coli*, *B. cereus* and *B. subtilis* (1×10^8^ CFU/mL in serum free cell culture medium; 1 mL/reaction) were incubated with J774A.1 cells (1×10^8^/well, with or without one hour LT pretreatment) in an antibiotic free condition. After 90-minutes of incubation, a cell membrane impermeable antibiotic gentamicin (10 µg/mL) was added to the medium for 30 minutes to eliminate the extracellular bacteria. Macrophage cells containing engulfed bacteria were washed with 1×phosphate-buffered saline (PBS), and lysed in 1% Triton-X 100, 1×PBS solution. The cell lysate was then plated on LB agar plates to determine the number of living bacteria (CFU) engulfed by macrophages.

### Analyses of MEK/MAPK pathways

For *in vivo* analysis, C57BL/6J mice were intravenously treated with various dosages of LT (50, 200, 400 µg/kg body weight, LF∶PA = 15∶85). Three hours after treatment, the splenic and peripheral mononuclear cells were isolated and purified using a Ficoll-Paque PLUS kit (GE healthcare, Singapore, Singapore) according to the manufacturers' instructions. The cell lysate was then subjected to MEK1 analysis, as indicated in the following methods. For *in vitro* analysis, J774A.1 cells (1×10^6^/well) were cultured on six-well dishes treated with various concentrations of LT for three hours with or without lipopolysaccharide (LPS, 0.1 µg/mL, Sigma-Aldrich, St. Louis, MO, USA) [39]. PA treatments (100 ng/mL) were used as negative controls. Cell lysate was separated by SDS-PAGE and then transferred to a nitrocellulose paper, whereupon Western blotting analysis was performed, as previously described [39]. The levels of MEK1 were measured by specific antibody (anti-NH_2_-terminal MEK1, Upstate Biotechnology, Lake Placid, NY, USA). The cellular MAPKs were measured by probing the antibodies against MAPK pathway kinases (p42/44, phospho-p42/44 [pp42/44], p38, and phospho-Jun kinase (JNK), Cell Signaling Technology, Danvers, MA; anti-pp38 Promega, Madison WI, USA). Jun kinase activity was measured by phosphorylation of c-Jun using a kit (Cell Signaling Technology, Danvers, MA, USA). The intensity of the blots was measured using Image J (version 1.32; National Institutes of Health, USA) software.

### Analysis of TNF-α production

Macrophage J774A.1 cells (2×10^5^) were treated with 0.1 µg/mL LPS and various concentrations of LT for three hours, in 96-well dishes. The cell culture medium was then collected from each well. The TNF-α levels produced by macrophage cells was determined using an enzyme-linked immunosorbent assay (ELISA) kit from eBioscience, (San Diego, CA, USA) according to the manufacturer's instructions.

### Phagocytosis analysis by flow cytometry

Phagocytosis analysis using fluorescent beads by flow cytometry was modified from previously described methods [16], [46]–[47]. Silica beads (mean diameter 1.1 µm, Bangs Laboratories, Fishers, IN, USA) [48] were opsonized with fluorescein-labeled rat anti-mouse immunoglobulin (Ig, 1 µg/mL, Jackson ImmunoResearch Laboratories) for 30 min at room temperature (25°C) in 1×PBS. After blocking with 5% bovine serum albumin (BSA) in 1×PBS for one hour, the beads (1×10^7^) were mixed with J774A.1 cells (1×10^6^, with or without one hour LT pretreatment) in 200 µL cell-culture medium. After two washes (PBS-albumin, 300 g, 10 min, 25°C), and fixation (2% paraformaldehyde in PBS, 30 min) the levels of phagocytosis were revealed by the florescent intensity of cell-engulfed beads, and could be identified using fluorescence flow cytometry (FACScalibur, BD Biosciences). Beads were distinguished from the J774A.1 cells according to their forward and side scatter characteristics (Fig. 3A vs. 3C; R1 region: J774A.1 cells). We then analyzed the fluorescent signals (beads) from the R1 regions (J774A.1 cells) to identify the relative level of phagocytosis (Fig. 3B, 3D, 3F, 3H, 3J, right panels). The J774A.1 cells were gated and 10,000 events acquired for each sample. The background fluorescence (<5%) was used to set the [left (L)/right (R)] border lines in Fig. 3B, 3D, 3F, 3H and 3J by beads-untreated J774A.1 cells. The level of phagocytosis/non-phagocytosis ratio in the beads was normalized to 100% by PA treated (LF untreated) J774A.1 cells. To avoid inefficient binding to cellular receptors [49], all LT treatments were supplied at the same doses levels as PA (85 ng/mL).

### Cell death analysis by flow cytometry

Following the previously described method [50], the dead cell subpopulations was determined by staining the cells with allophycocyanin (APC) conjugated annexin V (annexin V-APC) and propidium iodide (PI). The macrophage J774A.1 cells were treated with PA or various doses of LT for 3 hours. After the treatments, the total number of cells (5×10^5^ per sample) was washed twice with PBS for staining. The cells were stained by annexin V-APC (0.5 µg/ml) and PI (2 µg/ml) in the staining buffer (10 mM HEPES [4-(2-hydroxyethyl)-1-piperazineethanesulfonic acid], pH 7.4, 150 mM NaCl, 5 mM KCl, 1 mM MgCl_2_, 1.8 mM CaCl_2_) at room temperature for 20 min in the dark. After labeling, the cells were diluted in 10 mM HEPES buffer to a final volume of 600 µl, and analyzed by flow cytometry.

### ELISA analysis for circulating LT in mouse plasma

A sandwich ELISA modified from previously described methods [27] was performed. To investigate the turnover of LT in the blood circulation, mice were treated with 50 µg/kg body weight LT (LF∶PA = 15∶85). Blood specimens were collected from the mice 2 hours and 24 hours after the treatment, and mixed with the anticoagulant acid citrate dextrose (5∶1) (ACD: 20 mmol/L citric acid, 110 mol/L sodium citrate, and 5 mmol/L glucose; pH 7.3) to avoid blood clotting. Anti-PA and anti-LF antibodies were purified with serum from rabbits and mice immunized with high-performance liquid chromatography (HPLC)-purified PA or LF (6 cycles at 3-week intervals; 100 µg each cycle) as previously described [39]. Protein A column-purified polyclonal rabbit anti-PA or anti-LF antibodies (50 µl, 40 µg/ml) were incubated overnight in a 96-well ELISA plate (Corning Inc., Corning, NY, U.S.A.) at 4°C. The plates were then blocked with 5% bovine serum albumin (BSA; Sigma)/PBS for 2 h at room temperature. The plates were washed three times with 0.5% Tween 20-1× PBS and once with 1× PBS. To establish the standard cures, quantified (HPLC-purified *B. anthracis*-derived) native PA and LF were diluted with mouse serum at 1 µg/ml and serially diluted onto the ELISA plate. To detect PA and LF in blood of infected animals, serum was added to the plate initially diluted 1∶1 in 5% BSA-PBS, and serially diluted across the plate. After one-hr incubation, the plates were washed as described above. Protein A column-purified mouse anti-PA or anti-LF polyclonal antibodies were diluted 1∶1000 in 5% BSA-PBS and added to the plate for one-hr of incubation. The plate was then washed again, followed by the addition of goat anti-mouse immunoglobulin G (IgG) (diluted in 5% BSA-PBS). After one hour of incubation and two washes (PBS), donkey anti-goat horseradish peroxidase (HRP) conjugate (minimal cross-reactivity to mouse and rabbit IgGs, Jackson ImmunoResearch Laboratories, West Grove, PA, USA) was diluted in 5% BSA-PBS and incubated for additional one hour. ELISA reactions were developed with the substrate o-phenylenediamine (OPD) (Sigma). Experiments were run in triplicate, averaging each data point with standard deviation.

### Septic shock mouse model

Following the previously described method [51], fresh *E. coli* culture was collected when the OD reached 0.4–0.5. Using a predetermined standard curve [51], we adjusted the *E. coli* suspension to 2×10^7^ CFU/mL in normal saline. C57BL/6J mice received sequential intravenous treatments of *E. coli* (2×10^9^ to 5×10^9^ CFU/kg) and LT (50 µg/kg to 400 µg/kg). The mortality of the mice was recorded, and blood specimens were collected from the mice subjected to combined treatments of *E. coli* (2×10^9^ CFU/kg) and LT (50 µg/kg) at 2, 16, and 24 hours. Because mortality occurred within 24 hours (Fig. 5C, day1 groups), the data of the LT-treated 24 hour groups (Fig. 5D, LT + *E. coli*, 24 hr groups), was obtained from the survivors. Blood samples were mixed with anticoagulant ACD (2∶1), and the blood/ACD mixture (15 µl) was applied directly to LB agar plates. The number of cells formed in the colony after an overnight culture was recorded.

### Statistical analysis

All results were calculated from data of no fewer than three independent experiments. Statistical differences between groups were calculated using Student t test and presented as mean ± standard deviation (SD). A *P* value of less than 0.05 (*P*<0.05) was considered statistically significant. The statistical tests were carried out and output to graphs using Microsoft Excel (Microsoft Taiwan, Taipei, Taiwan) and SigmaPlot (Systat Software, Point Richmond, CA, USA) software.
